# Using a multi-omic approach to investigate the mechanism of 12-bis-THA activity against *Burkholderia thailandensis*

**DOI:** 10.3389/fmicb.2022.1092230

**Published:** 2023-05-12

**Authors:** Adam Pattinson, Sandeep Bahia, Gwénaëlle Le Gall, Christopher J. Morris, Sarah V. Harding, Michael McArthur

**Affiliations:** ^1^Norwich Medical School, Bob Champion Building for Research and Education, University of East Anglia, Norwich, United Kingdom; ^2^School of Pharmacy, University of East Anglia, Norwich, United Kingdom; ^3^CBR Division, Defense Science and Technology Laboratory, Salisbury, United Kingdom; ^4^Department of Respiratory Sciences, University of Leicester, Leicester, United Kingdom

**Keywords:** antimicrobial, mechanism-of-action, proteomic, metabolomics, respiration, ATP synthase

## Abstract

*Burkholderia pseudomallei* is the causative agent of the tropical disease, melioidosis. It is intrinsically resistant to many antimicrobials and treatment requires an onerous regimen of intravenous and orally administered drugs. Relapse of disease and high rates of mortality following treatment are common, demonstrating the need for new anti-*Burkholderia* agents. The cationic bola-amphiphile, 12,12′-(dodecane-1,12-diyl) bis (9-amino-1,2,3,4-tetrahydroacridinium), referred to as 12-bis-THA, is a molecule with the potential to treat *Burkholderia* infections. 12-bis-THA spontaneously forms cationic nanoparticles that bind anionic phospholipids in the prokaryotic membrane and are readily internalized. In this study, we examine the antimicrobial activity of 12-bis-THA against strains of *Burkholderia thailandensis*. As *B. pseudomallei* produces a polysaccharide capsule we first examined if this extra barrier influenced the activity of 12-bis-THA which is known to act on the bacterial envelope. Therefore two strains of *B. thailandensis* were selected for further testing, strain E264 which does not produce a capsule and strain E555 which does produce a capsule that is chemically similar to that found in *B. pseudomallei*. In this study no difference in the minimum inhibitory concentration (MIC) was observed when capsulated (E555) and unencapsulated (E264) strains of *B. thailandensis* were compared, however time-kill analysis showed that the unencapsulated strain was more susceptible to 12-bis-THA. The presence of the capsule did not affect the membrane permeation of 12-bis-THA at MIC concentrations. Proteomic and metabolomic analyses showed that 12-bis-THA causes a shift in central metabolism away from glycolysis and glyoxylate cycle, and suppressed the production of the F_1_ domain of ATP synthase. In summary, we provide insight into the molecular mechanisms underpinning the activity of 12-bis-THA against *B. thailandensis* and discuss its potential for further development.

## 1. Introduction

*Burkholderia pseudomallei* is the causative agent of melioidosis, a disease of the tropics and sub-tropics which causes approximately 165,000 cases and 89,000 deaths annually (Limmathurotsakul et al., 2016). Bacterial transmission can occur through ingestion and inhalation however sub-cutaneous inoculation through contact with contaminated water and soil is thought to be the most common route of infection (Limmathurotsakul and Peacock, 2011). Clinical manifestation is highly diverse as melioidosis can present as an asymptomatic latent infection (Johnson and Ali, 1990), a chronic infection comprised of multiple localized abscesses (Goel et al., 2016), or as an acute infection, consisting of necrotizing pneumonia or fulminating sepsis which can rapidly lead to death (Currie et al., 2000).

The mortality rate of melioidosis is reduced by rapid diagnosis and appropriate antimicrobial intervention (Currie et al., 2010; Limmathurotsakul et al., 2010). The current treatment regime uses a biphasic approach consisting of an acute phase and an eradication phase (Dance, 2014). During the acute phase, carbapenems (meropenem) or 3^rd^ generation cephalosporins (ceftazidime) are delivered intravenously for 10 days or more, to prevent death from overwhelming sepsis. After successful acute treatment, the patient enters the eradication phase which consists of oral co-trimoxazole or co-amoxiclav for 3–6 months to kill residual bacteria and reduce the risk of relapse. The current treatment regimen is reliant on strict patient adherence during the eradication phase to limit the chance of recurrence, however side effects from co-trimoxazole, which occur in 39% of recipients (Chetchotisakd et al., 2014), reduce patient compliance. Additionally, while resistance to meropenem (Price et al., 2017), co-trimoxazole (Wuthiekanun et al., 2005), and ceftazidime (Rao et al., 2019) are low, cases are increasingly being reported. Due to this, there is an ongoing need for novel antimicrobials that can be used as post-exposure prophylaxis or new treatment strategies.

Research into new antimicrobials to treat *B. pseudomallei* is partially limited by its classification as a Containment Level 3 organism, meaning that it has the potential to cause serious human disease. Due to its high aerosol infectivity and a high degree of antimicrobial resistance, it is classified as a tier 1 select agent by the Center for Disease Control and Prevention, and as a category B biothreat agent by the National Institute of Allergy and Infectious Diseases, which emphasizes the importance of identifying improved treatments. Preliminary research routinely uses *Burkholderia thailandensis* as a surrogate organism as it is a member of the *B. pseudomallei* complex, a group of phenotypically similar but genetically distinct organisms. Unlike *B. pseudomallei*, *B. thailandensis* is typically avirulent in humans, although it shares a high degree of genomic (Yu et al., 2006) and phenotypic similarity including antimicrobial resistance profiles (Amiss et al., 2020). Most *B. thailandensis* strains lack a polysaccharide capsule which is an important virulence factor for *B. pseudomallei* (Reckseidler et al., 2001) and provides some protection from host complement (Reckseidler-Zenteno et al., 2005). *B. thailandensis* strain E555 has gained a genomic insert containing genes encoding for the manufacture and transport of a polysaccharide capsule (Sim et al., 2010) that is similar to the one found in *B. pseudomallei* (Bayliss et al., 2017) making it a good surrogate for the screening of new candidates to treat melioidosis.

One type of molecule that has potential as a new antimicrobial to treat *Burkholderia* infections are cationic bola-amphiphiles, such as the recently described 12,12′-(dodecane-1,12-diyl) bis(9-amino-1,2,3,4tetrahydroacridinium), known as 12-bis-THA (Marín-Menéndez et al., 2017; Montis et al., 2020). It is a symmetrical molecule with two planar headgroups and a delocalized cationic charge linked by an aliphatic chain. Hence, they readily form nanoparticles in aqueous solutions that bind to anionic lipids within the bacterial membrane, such as cardiolipin (Marín-Menéndez et al., 2017). Membrane binding leads to internalization and the nanoparticles can carry oligonucleotide antimicrobial cargo into the bacterial cytoplasm (Montis et al., 2020). A likely consequence of binding to cardiolipin is the disruption of membrane-bound respiratory chains, with a concomitant effect on proton motive force and certain respiratory complexes (Feng et al., 2015) to inhibit bacterial growth. In this study, we combine MIC and time-kill assays with membrane permeabilization studies and confocal microscopy to investigate the antimicrobial activity of 12-bis-THA against capsulated and unencapsulated strains of the surrogate organism *B. thailandensis*. The data suggested that lysis was not the mechanism of action at MIC concentrations, therefore we used integrated proteomics and Nuclear Magnetic Resonance (NMR)-based metabolomics to provide insight into the mechanisms of action of 12-bis-THA against *B. thailandensis* strain E555. These studies suggested the antimicrobial effect was linked to the suppression of bacterial respiratory complexes and glycolysis.

## 2. Materials and methods

### 2.1. Synthesis of 12-bis-THA

[12-bis-THA]Cl_2_ was obtained by anion exchange of [12-bis-THA]I_2_ (synthesized by Shanghai Chempartner Co., Ltd). Briefly, a mixture of 1,2,3,4-tetrahydroacridin-9-amine and the 1,12-diiododecane (2:1 molar ratio) was stirred at 155°C for 5 h, then cooled down. The crude product was triturated with 90% ethanol (ethanol: deionized water, V:V = 90:10) 3 times at reflux to get the iodide salt of the desired compound. Ion exchange of [12-bis-THA]I_2_ with chloride was carried out using chloride form of Dowex resin (16–100 mesh) from Sigma-Aldrich previously conditioned with HCl 5%. Dowex resin was washed on a Buchner funnel with Milli-Q water (1 L), then it was conditioned with HCl 5% (0.5 L) and rinsed again with water Milli-Q until neutral pH had been achieved. Additionally, washing was continued until no chloride precipitation from the eluate was detected following treatment with AgNO_3_. Finally, the resin was thoroughly washed with methanol (1 L). Following the activation of the resin, [12-bis-THA]I_2_ (100 mg, 0.12 mmol) was dissolved in methanol (100–120 ml) and mixed with Dowex (10 g of wet resin in methanol). The mixture was left stirring at room temperature overnight, then it was filtered under pressure through a size 4 Buchner filter, dried and stored at −20°C under N_2_ in the dark.

### 2.2. Minimum inhibitory concentration assays

12-bis-THA was tested to determine their Minimum Inhibitory Concentration (MIC) against *B. thailandensis* E264 and *B. thailandensis* E555 using the broth microdilution method in accordance with CLSI guidelines. Bacterial colonies were isolated from a Luria-Bertani agar plate and suspended in 1X phosphate buffered saline (PBS) to a turbidity equivalent to a 0.5 McFarland standard. The bacterial suspension was then diluted 1:500 into 2X Cation Adjusted Muller-Hinton Broth (CAMHB; Fisher Scientific, United Kingdom) and vortexed to disaggregate bacterial clumps. 100 μl of the bacterial culture was transferred to the wells of a 96-well plate containing the different treatments. Treatments were prepared by serially diluting compounds 2-fold in sterile water in a 96-well plate to produce a final volume of 100 μl (total 200 μl for treatments and bacterial culture). Plates were incubated at 37°C with agitation for 18 h and the MIC was defined as the lowest concentration that caused an absence of visible growth.

### 2.3. Time-kill study

An overnight culture was prepared by suspending a single bacterial colony from a streak agar plate into 1X CAMHB which was incubated overnight at 37°C with shaking at 180 rpm. The bacterial culture was diluted to an optical density (O.D_600_) of 1.0 in 1X PBS and then diluted 1:1000 (~1 × 10^5^ colony-forming units per milliliter (CFU/ml) into prewarmed 1X CAMHB containing treatments. Bacterial cultures were then incubated for 24 h at 37°C with shaking at 200 rpm. To measure the antimicrobial activity of 12-bis-THA against *B. thailandensis* strains E264 and E555, 20 μl aliquots of treated bacteria were taken at 0, 3, 6, and 24 h and serially diluted 10-fold in 180 μl of 1X PBS. The bacterial inoculum was spot plated into LB agar and incubated at 37°C for 24 h before enumeration. Bactericidal activity was defined as a >3 log_10_ reduction in CFUs when compared to the starting inoculum.

### 2.4. Confocal laser scanning microscopy

An overnight culture of *B. thailandensis* strain E264 or E555 was diluted 2% v/v into 1X CAMHB and incubated at 37°C and 200 rpm. Bacterial cultures were grown to mid-log phase (0.3–0.5 OD_600_) and then diluted by transferring 500 μl of bacteria into an Eppendorf tube containing either 500 μl of 12-bis-THA particles (100 μg/ml) or 500 μl of PBS. *B. thailandensis* was then incubated with 12-bis-THA particles for 90 min at 37°C with agitation. Bacteria were stained for a further 30 min using the lipophilic dye, FM4-64 FX (Thermo Fisher Scientific, United Kingdom; 1 mg/ml stock solution) by transferring 10 μl of dye.

Bacterial cultures were pelleted at 4000 × *g* for 5 min and resuspended in 100 μl of PBS. 100 μl of bacteria were smeared onto a slide and left to incubate at room temperature in the dark for 30 min. Slides were gently rinsed with 1X PBS to remove the loosely attached bacteria and left to dry for 5 min at 37°C in the dark. Coverslips were mounted using Fluoromount aqueous mounting media (Sigma-Aldrich, Germany) before imaging. Slides were imaged using a Zeiss LSM800 confocal microscope (Zeiss, Germany). 12-bis-THA particle fluorescence was captured using the Hoechst H3258 emission filter at γ_ex_ 405 nm and γ_em_ 455 nm and FM4-64 FX was captured at γ_ex_ 515 nm and γ_em_ 630 nm.

### 2.5. Membrane permeability assay

An overnight culture of *B. thailandensis* strain E264 or E555 was diluted to 0.1 OD_600_ in 1X CAMHB and grown to 0.5 OD_600_ at 37°C and 200 rpm. 900 μl of bacterial cultures were transferred to Eppendorf tubes containing 100 μl of 12-bis-THA in water to produce the final concentrations 0, 20, 40, 60 or 80 μg/ml. Cultures were incubated for 1 h at 37°C with shaking at 200 rpm. The bacterial cultures were then centrifuged at 4000 × *g* and 4°C for 20 min and the supernatant was collected. The lactate dehydrogenase (LDH) activity of the bacterial supernatant was determined as per the manufacturer’s guidelines (Thermo Fisher Scientific, United Kingdom) with the exception that the reaction was left overnight at 37°C prior to spectrophotometric analysis at 490 nm (FLUOstar Omega Microplate Reader – BMG Labtech).

### 2.6. Microbiological growth analysis

An overnight culture of *B. thailandensis* strain E555 was diluted to an OD_600_ of 0.05 in 100 ml of Luria-Bertani broth and grown at 37°C with shaking at 200 rpm. At 0.5 OD_600_ the culture was split into three 10 ml aliquots that were challenged with either distilled water (untreated), 0.8 or 8 μg/ml of 12-bis-THA. Bacteria were incubated for 2.5 h at 37°C following challenge. Cultures were centrifuged at 6000 × *g* and 4°C, and pellets washed twice with chilled 1X PBS. Following the final wash, the supernatant was discarded, and the pellet was stored at-80°C prior to protein, and metabolite extraction.

### 2.7. Protein extraction and quantification

Bacterial pellets were suspended in 500 μl of lysis buffer (8 M urea and 25 mM NaHCO_3_) and sonicated at 4°C using a Bioruptor Plus set to “high” frequency. The sample was then spun at 16,100 × *g* for 40 min at room temperature. Following centrifugation, the supernatant was retained, and the pellet discarded. Protein concentration was determined using the Pierce™ BCA Protein Assay Kit (Thermofisher). Liquid protein extract was sent to the Proteomics facility at the University of Bristol for LC–MS/MS analysis and tandem mass tagging (TMT).

### 2.8. TMT Labelling and high pH reversed-phase chromatography

Aliquots of 25 μg of each sample were digested with trypsin (2.5 μg trypsin per 100 μg protein; 37°C, overnight), labelled with Tandem Mass Tag (TMT) 10 plex reagents according to the manufacturer’s protocol (Thermo Fisher Scientific, Loughborough, United Kingdom) and the labelled samples pooled. These were evaporated to dryness, resuspended in 5% (vol/vol) formic acid and desalted using a SepPak cartridge according to the manufacturer’s instructions (Waters, Milford, Massachusetts, United States). Eluate from the SepPak cartridge was again evaporated to dryness and resuspended in buffer A (20 mM ammonium hydroxide, pH 10) prior to fractionation by high pH, reversed-phase chromatography using an Ultimate 3,000 liquid chromatography system (Thermo Scientific). In brief, the sample was loaded onto an XBridge BEH C18 Column (130 Å, 3.5 μm, 2.1 mm × 150 mm, Waters, United Kingdom) in buffer A and peptides eluted with an increasing gradient of buffer B (20 mM ammonium hydroxide in acetonitrile, pH 10) from 0 to 95% over 60 min. The resulting fractions were evaporated to dryness and resuspended in 1% (v/v) formic acid prior to analysis by nano-LC MS/MS using an Orbitrap Fusion Lumos mass spectrometer (Thermo Fisher Scientific, United Kingdom).

### 2.9. Nano-LC mass spectrometry

High pH RP fractions were further fractionated using an Ultimate 3,000 nano-LC system in line with an Orbitrap Fusion Lumos mass spectrometer (Thermo Fisher Scientific, UK). In brief, peptides in 1% (vol/vol) formic acid were injected onto an Acclaim PepMap C18 nano-trap column (Thermo Fisher Scientific, United Kingdom). After washing with 0.5% (v/v) acetonitrile and 0.1% (v/v) formic acid, peptides were resolved on a 250 mm × 75 μm Acclaim PepMap C18 reverse phase analytical column (Thermo Fisher Scientific, United Kingdom) over a 150 min organic gradient, using seven gradient segments (1–6% solvent B over 1 min, 6–15% B over 58 min, 15–32% B over 58 min, 32–40% B over 5 min, 40–90% B over 1 min, held at 90% B for 6 min and then reduced to 1% B over 1 min) with a flow rate of 300 nl min^−1^. Solvent A was 0.1% formic acid and Solvent B was aqueous 80% acetonitrile in 0.1% formic acid. Peptides were ionized by nano-electrospray ionization at 2.0 kV using a stainless-steel emitter with an internal diameter of 30 μm (Thermo Fisher Scientific, UK) and a capillary temperature of 275°C.

All spectra were acquired using an Orbitrap Fusion Lumos mass spectrometer controlled by Xcalibur 4.1 software (Thermo Fisher Scientific, UK) and operated in data-dependent acquisition mode using an SPS-MS3 workflow. FTMS1 spectra were collected at a resolution of 120,000 with an automatic gain control (AGC) target of 200,000 and a max injection time of 50 ms. Precursors were filtered with an intensity threshold of 5,000, according to charge state (to include charge states 2–7) and with monoisotopic peak determination set to Peptide. Previously interrogated precursors were excluded using a dynamic window (60 s +/−10 ppm). The MS2 precursors were isolated with a quadrupole isolation window of 0.7 m/z. ITMS2 spectra were collected with an AGC target of 10,000 max injection time of 70 ms and CID collision energy of 35%.

For FTMS3 analysis, the Orbitrap was operated at 50,000 resolution with an AGC target of 50,000 and a max injection time of 105 ms. Precursors were fragmented by high energy collision dissociation (HCD) at a normalized collision energy of 60% to ensure maximal TMT reporter ion yield. Synchronous Precursor Selection (SPS) was enabled to include up to 5 MS2 fragment ions in the FTMS3 scan.

### 2.10. Data analysis

The raw data files were processed and quantified using Proteome Discoverer software v2.1 (Thermo Scientific) and searched against the UniProt *B. thailandensis* strain E264 database (downloaded August 2019: 5565 entries) using the SEQUEST algorithm. Peptide precursor mass tolerance was set at 10 ppm, and MS/MS tolerance was set at 0.6 Da. Search criteria included oxidation of methionine (+15.9949) as a variable modification and carbamidomethylation of cysteine (+57.0214) and the addition of the TMT mass tag (+229.163) to peptide N-termini and lysine as fixed modifications. Searches were performed with full tryptic digestion and a maximum of two missed cleavages were allowed. The reverse database search option was enabled and all data was filtered to satisfy a false discovery rate (FDR) of 5%. Data handling and analysis were performed using Perseus (48). Reverse hits and contaminants were removed and missing values were assigned before performing statistical analysis. The intensity data were transformed to log2 and filtered to contain at least three values. Statistical significance was determined by performing Student’s t-tests with a Benjamini-Hochberg *post-hoc* test to correct for multiple hypothesis testing. Volcano plots were produced using the statistical parameters FDR of 0.01 and an S0 value of 1. Perseus was also used to perform principle component analysis (PCA) clustering.

### 2.11. Metabolite extractions

The metabolome of *B. thailandensis* was extracted as described by Moreira et al. (31). Briefly, glass beads were added to the bacterial pellets, which were retained from the microbial growth assay, and resuspended in 600 μl of 80°C ethanol (75% v/v). Samples were vortexed for 30 s and incubated at 80°C for 3 min. The samples were vortexed again for 30 s. The supernatant was collected by centrifugation at 10,000 × *g* and 4°C for 10 min. This process was repeated. The supernatant was dried under vacuum using a Savant SC210A SpeedVac Concentrator and the extracts were stored at −20°C until analysis.

### 2.12. Metabolome and data analysis

The dried extracts were prepared by dissolving pellets in 0.6 ml NMR buffer (100 ml D_2_O containing 0.26 g NaH_2_PO_4_, 1.41 g K_2_HPO_4_, and 1 mM deuterated trimethyl silylpropionate (TSP) as a reference compound). The spectra were recorded using a Bruker Avance NEO 600 MHz NMR spectrometer equipped with TCI CryoProbe. The data were collected at 25°C using TSP as a reference compound and processed using the TopSpin version 3.2 software package. The metabolites were quantified using the software Chenomx NMR Suite 7.0. Metaboanalyst was used to perform PCA clustering.

### 2.13. Statistical analysis

Graphpad Prism version 5.0 was used to perform statistically analysis on data from bacterial culture experiments and changes in metabolite concentrations. A one-way ANOVA with a Dunnets *post-hoc* test was performed to determine significant difference in metabolite concentration. Two-way ANOVAs were performed to investigate statistical differences in the time-kill, membrane permeabilisation and growth curve analysis. Significance was denoted using *p-value < 0.05; **p-value < 0.01; ***p-value < 0.001.

## 3. Results

### 3.1. Antimicrobial properties of 12-bis-THA

Antimicrobial susceptibility testing was performed to determine the activity of 12-bis-THA against *B. thailandensis* strains E264 and E555. The MIC values for both strains, determined by broth microdilution assay, were similar and ranged between 2 and 8 μg/ml.

**Figure 1:**
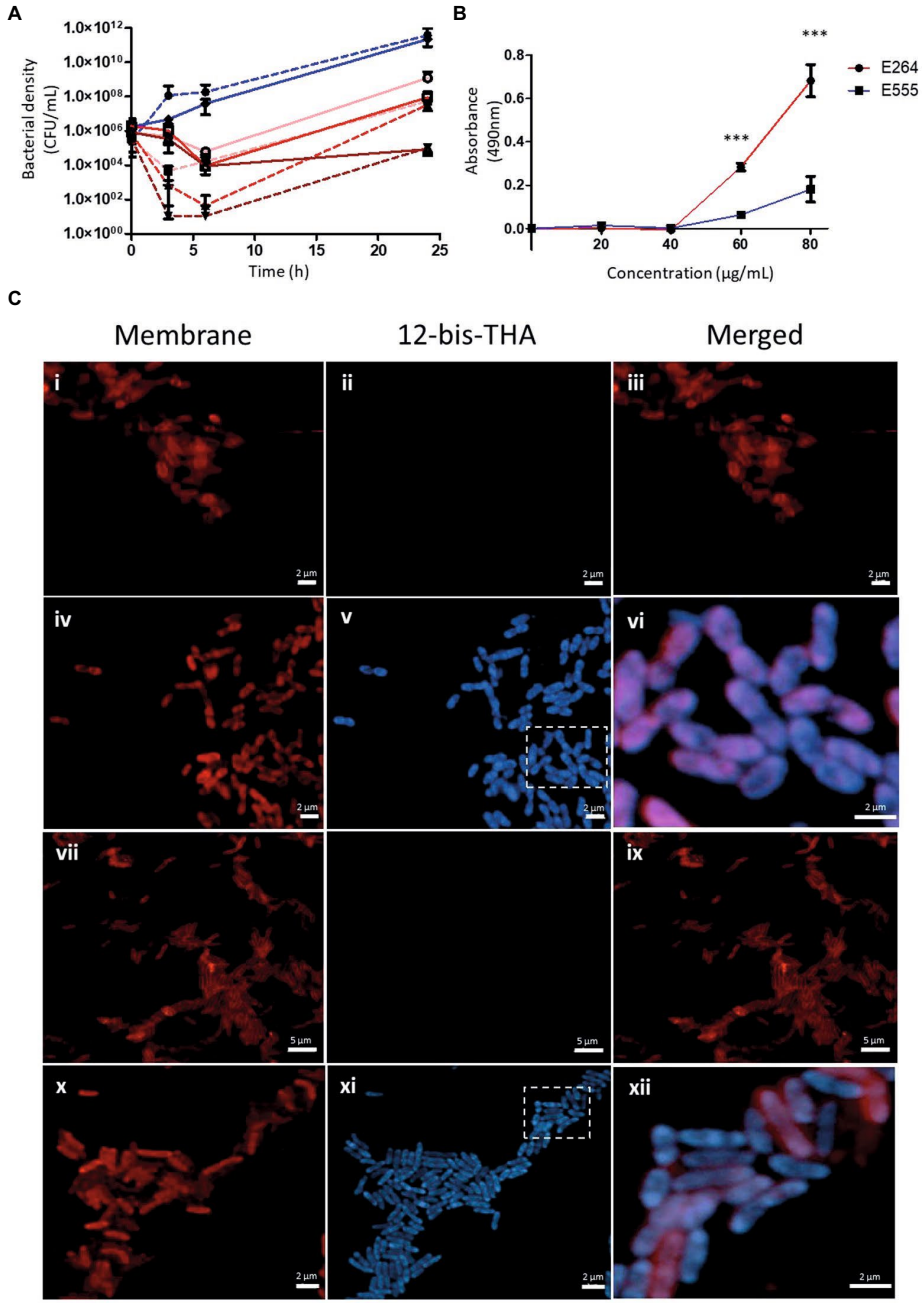
The antimicrobial action of 12-bis-THA against capsulated and unencapsulated strains of *B. thailandensis*. **(A)** Time-kill study of 2, 4 and 8 μg/ml of 12-bis-THA against *B. thailandensis* strain E264 (dashed line) or E555 (solid line). Blue lines denote untreated bacteria and those incubated with 2, 4 and 8 μg/ml of 12-bis-THA by pink, red and brown lines, respectively. Error bars show standard deviation. **(B)** Membrane permeabilisation of E264 (red) and E555 (blue) in response to 20, 40, 60 or 80 μg/ml of 12-bis-THA. Confocal micrographs in **(C)** show untreated E264 **(i–iii)** and E555 **(vii–ix)** cells, or cells treated with 12-bis-THA: E264 **(iv–vi)** and E555 **(x–xii)**. Membranes (γ_ex_ 515 nm/γ_em_ 630 nm) and 12-bis-THA (γ_ex_ 405 nm/γ_em_ 455 nm) were imaged with a Zeiss LSM 800 confocal microscope. Areas contained within the box were magnified in the merged image. Error bars for **(i–iii)** show 2 μm whereas **(iv–vi)** show 5 μm.

Time-kill studies showed *B. thailandensis* strain E264 was more sensitive to 12-bis-THA, which exhibited bactericidal activity at 4 and 8 μg/ml (Figure 1A). At 3 h, 2 and 4 μg/ml caused a 1.8 and 2.7 log_10_ reduction in bacterial density whereas 8 μg/ml reduced the bacterial density to below the limit of detection. Following 6 h of incubation, 2 μg/ml of 12-bis-THA caused a 1.3 log_10_ reduction in bacterial density. At this time point both 4 and 8 μg/ml reduced the viability of *B. thailandensis* E264 cultures below the limit of detection. Conversely, E555 was less sensitive to 12-bis-THA following 3 h of exposure irrespective of the concentration as bacterial densities were reduced by only 0.2, 0.3, and 0.4 log_10_ at 2, 4, and 8 μg/ml, respectively. Bacterial killing increased at 6 h as bacterial cultures were reduced by 1.32, 2.33, and 2.00 log_10_ CFU/ml at 2, 4, and 8 μg/ml, respectively. At 24 h, both strains displayed bacterial regrowth including those that had were previously below the limit of detection, which may be due to 12-bis-THA particles aggregating and precipitating from the media at the 24 h timepoint. These data suggest that the polysaccharide capsule may provide protection from 12-bis-THA particles interacting with the cell wall.

12-bis-THA is known to interact with prokaryotic anionic phospholipids such as cardiolipin (Marín-Menéndez et al., 2017) and LPS (Montis et al., 2020). Studies in artificial membranes supplemented with these lipids have shown that incubation with 12-bis-THA can lead to membrane destabilization and lysis (Montis et al., 2020). Considering this, we investigated membrane permeabilisation following exposure to 12-bis-THA to determine if they caused cytoplasmic leakage in *B. thailandensis*. The enzymatic activity of LDH, a cytoplasmic enzyme, was measured within the supernatant of E264 and E555 cultures that had been incubated with 20, 40, 60, or 80 μg/ml of 12-bis-THA (Figure 1B). The data showed that no LDH activity was detected in the supernatants of either strain following incubation with 20 or 40 μg/ml. Significant increases in concentration-dependent LDH activity were observed following incubation with 60 μg/ml (*p*-value < 0.001) or 80 μg/ml (*p*-value < 0.001) of 12-bis-THA. However this effect was more notable in the unencapsulated strain compared to the encapsulated strain. As permeabilisation was only observed at high concentrations of 12-bis-THA, it was concluded that lysis is unlikely at MIC concentrations. Hence, CLSM was used to investigate further the effect of 12-bis-THA on the bacterial cell structure.

Bacteria grown to an OD_600_ of 0.3 (~10^9^ CFU/ml) were incubated with 50 μg/ml of 12-bis-THA for 120 min and the cytoplasmic membrane labelled with FM4-64 dye to distinguish between the interior and exterior of the cell. The FM4-64 staining of untreated E264 (Figures 1Ci–iii) and E555 (Figures 1Cvii–ix) showed the expected staining of the bacterial membrane (visible in the red channel) but not the cytoplasm which remained unstained. Treatment with 12-bis-THA (visible in the blue channel) with bacterial cells labelled with FM4-64 demonstrated intracellular penetration of the dye in E264 (Figures 1Civ–vi) and E555 (Figures 1Cx–xii), presumably due to the action of 12-bis-THA. The merged images showed that 12-bis-THA was predominantly within the cytoplasm with some signal observed within the membrane. All bacterial cells in view showed penetration of 12-bis-THA, suggesting that the capsule of E555 did not inhibit the process. No cell debris or a reduction in bacterial density was observed in any of the conditions, in comparison to the untreated controls.

### 3.2. Proteomic analysis of bacterial response to 12-bis-THA particles

**Figure 2:**
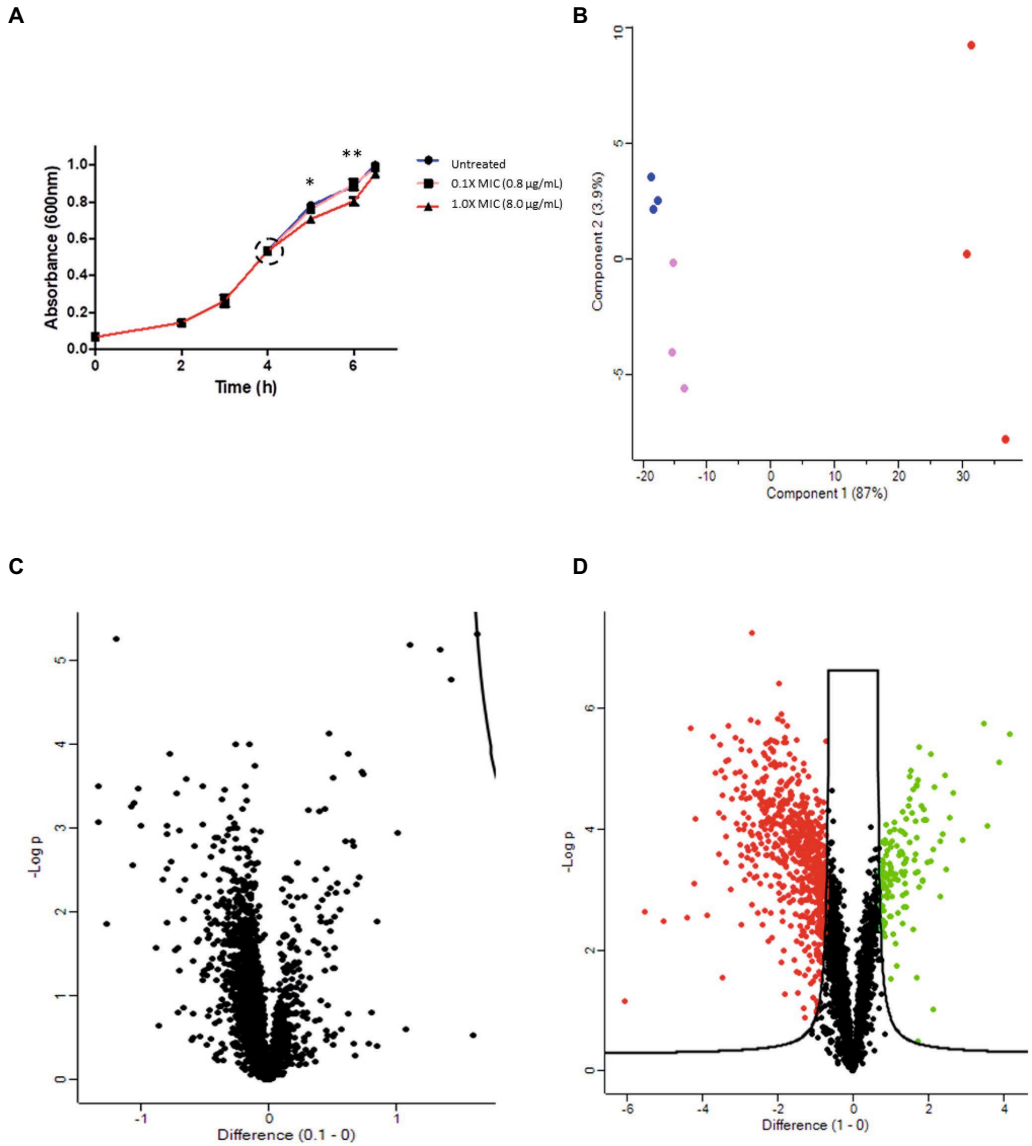
Proteomic analysis of the antimicrobial activity of 12-bis-THA against *B. thailandensis* E555. **(A)** The effect of 12-bis-THA on the growth kinetics of *B. thailandensis* at 0.1X MIC (0.8 μg/ml) and 1X (8 μg/ml) concentrations. Error bars show standard deviation and statistical significance was determined using a two-way ANOVA with a Benjamini-Hochberg *post-hoc* test where *p < 0.05 and **p < 0.01. **(B)** PCA analysis showing the separation of the *B. thailandensis* proteomes over the first two components. Green, pink and red data points represent the proteomes of untreated bacteria, those treated with 0.1X MIC or those treated with 1X MIC, respectively. Volcano plots of differentially abundant proteins in response to **(C)** 0.1X MIC or **(D)** 1 X MIC determined using the parameters FDR: 0.01 and S0: 1. Red and green data points show a decrease, and increase in protein abundance, respectively.

A proteomic study was performed to assess the antimicrobial activity effect of 12-bis-THA on actively growing *B. thailandensis* E555 cells. Samples were generated by incubating E555 cultures grown to mid-log (0.5 OD_600_) with 0.1X MIC (0.8 μg/ml) or 1X MIC (8.0 μg/ml) of 12-bis-THA for 2.5 h. No difference in the growth kinetics was observed when cultures were incubated with 0.1X MIC when compared to the untreated (water treated) control. Growth retardation was observed at 1 h (*p*-value < 0.05) and 2 h (*p*-value < 0.01) following incubation with 1X MIC of 12-bis-THA, however no significant difference was observed at the time of harvesting (Figure 2A). This was expected as samples were prepared with a high inoculum to produce enough material for the analyses, and the concentrations of 12-bis-THA were chosen to induce bacterial stress without causing death.

Protein extracts were analyzed using TMT LC–MS/MS for global proteomic analysis. A total of 3,150 proteins were detected, covering 56.6% of the *B. thailandensis* proteome, which is comparable to similar studies investigating the action of antimicrobials on this organism (Li et al., 2020). PCA of the proteomics data set showed a clear separation between the three groups (untreated, 0.1X MIC and 1X MIC) although the proteome of bacteria treated with 0.1X MIC clustered closely, with no overlap, to the untreated control. The proteome of cultures treated with 1X MIC separated far along principal component 1 suggesting considerable differences to the other groups (Figure 2B). To determine if there were significant changes in protein abundance following incubation with 12-bis-THA, volcano statistical analyses were performed. There was no difference between the control and the bacteria treated with 0.1X MIC which is consistent with the lack of effect on bacterial growth (Figure 2C). In contrast, 139 proteins significantly increased in abundance, and 671 significantly decreased in abundance in *B. thailandensis* when exposed to 1X MIC in comparison to the untreated control (Figure 2D).

### 3.3. Proteomic response of E555 central metabolism to 12-bis-THA

The proteomic data set was manually interrogated to identify pathways that were differentially abundant in response to 12-bis-THA treatment, compared to the untreated control. Analysis showed that 12-bis-THA caused changes in the abundance of proteins involved in central metabolism following exposure to 12-bis-THA. Those identified as significantly up-or down-regulated are shown in bold in Table 1.

**Table 1:** Differentially abundant proteins associated with central metabolism in *B. thailandensis* E555 following incubation with 12-bis-THA.

	Accession number	Protein name	Gene	Fold change (Log_2_)	
				0.1X MIC	1X MIC
Glycolysis	Q2SYG4	Phosphoglucomutase	BTH_I1489	−0.16	−0.39
	Q2T884	6-Phosphofructokinase	BTH_II0415	0.04	**−1.25**
	Q2SZ59	Phosphoglycerate mutase	BTH_I1243	0.17	0.36
	Q2SZL1	Phosphoglycerate mutase	BTH_I1085	−0.21	**−0.96**
β-oxidation	Q2T128	Acyl-CoA dehydrogenase domain-containing protein	BTH_I0564	0.002	**1.69**
	Q2T125	Enoyl-CoA hydratase	BTH_I0567	0.14	**1.00**
	Q2T127	3-Hydroxyacyl-CoA dehydrogenase	BTH_I0565	0.06	**1.89**
	Q2T126	Acetyl-CoA acetyltransferase	BTH_I0566	0.08	**1.57**
TCA cycle	Q2T7J2	Mdh1	BTH_II0658	0.07	−0.03
	Q2T4T8	Mdh2	BTH_II1671	−0.13	**−0.79**
	Q2T7I5	GltA	BTH_II0665	0.06	0.47
	Q2T7J6	AcnA	BTH_II0654	−0.04	0.04
	Q2T0I4	Icd	BTH_I0759	−0.07	0.14
	Q2SVH6	SucA	BTH_I2556	−0.03	**0.89**
	Q2SVH7	SucB	BTH_I2555	−0.08	**0.89**
	Q2T0U7	SucC	BTH_I0646	0.02	0.46
	Q2T0U6	SucD	BTH_I0647	−0.08	0.47
	Q2T7J0	Succinate dehydrogenase cytochrome b556 subunit	BTH_II0660	−0.05	−0.09
	Q2T7I9	Succinate dehydrogenase, hydrophobic membrane anchor protein	BTH_II0661	−0.16	0.21
	Q2T7I7	Succinate dehydrogenase iron–sulfur subunit	BTH_II0663	−0.02	0.07
	Q2T3N6	Tartrate/fumarate family Fe-S type hydro-lyase	BTH_II2020	−0.09	0.09
Glyoxylate bypass	Q2SX27	AceA	BTH_I1998	−0.24	**−1.33**
	Q2SX31	AceB	BTH_I1994	−0.13	−0.62
Respiratory complex III (Cytochrome C reductase)	Q2SUB8	PetA	BTH_I2977	0.04	**−2.03**
	Q2SUB9	Cytochrome B	BTH_I2976	−0.1	**−1.36**
	Q2SUC0	Cytochrome c1	BTH_I2975	−0.13	**−1.21**
Respiratory complex V (ATP synthase)	Q2T880	ATP synthase subunit beta	BTH_II0419	0.05	**−2.01**
	Q2T879	ATP synthase epsilon chain	BTH_II0420	0.06	**−1.29**
	Q2T874	ATP synthase subunit b	BTH_II0425	0.04	**−3.39**
	Q2T873	ATP synthase subunit alpha	BTH_II0426	0.11	**−1.69**
	Q2T872	ATP synthase F1 gamma subunit	BTH_II0427	0.08	**−2.97**

Values marked in bold show statistical significance that was determined using the volcano statistical parameters FDR: 0.01 and S0: 1.

Four proteins involved in glycolysis were identified and two were significantly downregulated [BTH_II0415: −1.25-fold (*p*-value < 0.01) and BTH_I1085: −0.96-fold (*p*-value < 0.01)]. Four proteins involved in β-oxidation increased in abundance (BTH_I0564: 1.69-fold (*p*-value < 0.01), BTH_I0565: 1.89-fold (*p*-value < 0.01), BTH_I0566: 1.57-fold (*p*-value < 0.01), BTH_I0567: 1.00-fold (*p*-value < 0.05). Two proteins involved with the tricarboxylic acid (TCA) cycle increased in abundance in response to 1.0X MIC 12-bis-THA. For example, the protein subunits of α-ketoglutarate dehydrogenase [SucA (*p*-value < 0.05) and SucB (*p*-value < 0.05)] were both increased by 0.89-fold. Conversely, AceA and AceB, enzymatic components of the glyoxylate cycle, but only AceA was downregulated 1.33-fold (*p*-value < 0.05) following incubation with 1.0X MIC of 12-bis-THA (Table 1).

1.0X MIC of 12-bis-THA caused the downregulation of protein components of respiratory complex III (Cytochrome C reductase) and respiratory complex V (ATP synthase; Table 1). Three proteins from Cytochrome C reductase were identified in the proteomics data set and all were downregulated. PetA, which contains the Rieske center (Luo et al., 2013), was significantly downregulated 2.03-fold (*p*-value < 0.001). Cytochrome c1, the sequential next step in trafficking electrons to Cytochrome C, was downregulated 1.21-fold (*p*-value < 0.01). Cytochrome B, which is a component of the Q cycle, was also significantly downregulated 1.36-fold (*p*-value < 0.01). Twelve proteins of the ATP synthase complex were identified and five were significantly downregulated. ATP synthase subunit b was the only component of the ATP synthase F_0_ significantly downregulated and was reduced 3.39-fold (*p*-value < 0.001). All components of the F_1_ domain were downregulated apart from the δ subunit. The α and β subunits were significantly downregulated 1.69 (*p*-value < 0.01) and 2.01-fold (*p*-value < 0.001) respectively, whereas the γ and ε subunits were significantly downregulated 2.97 (*p*-value < 0.01) and 1.29-fold (*p*-value < 0.01) respectively. It was concluded that the proteomics data set suggests that 12-bis-THA induces metabolic remodeling in *B. thailandensis.*

### 3.4. Metabolomic analysis of *Burkholderia thailandensis* E555 in response to 12-bis-THA

**Figure 3:**
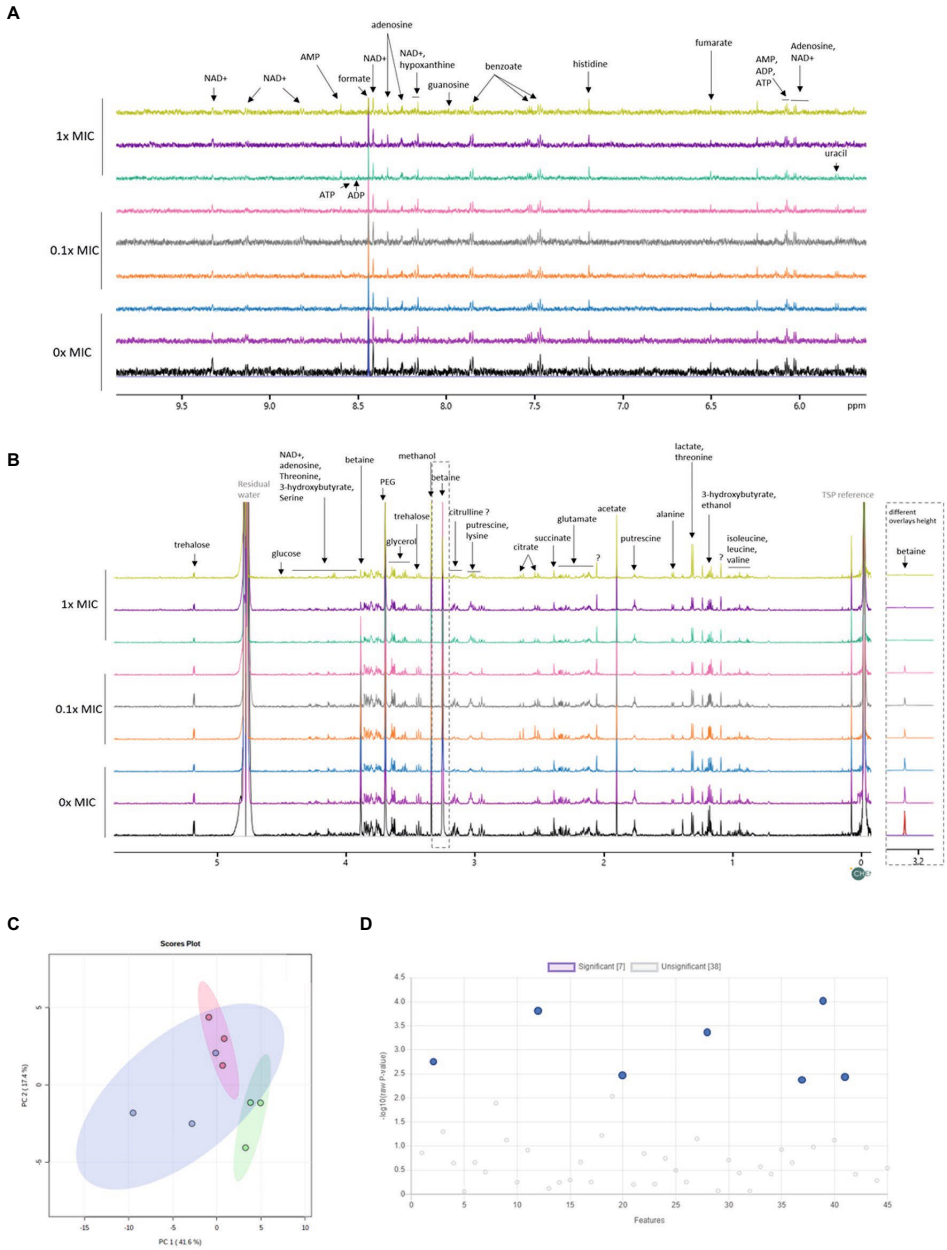
Changes in the metabolome of *B. thailandensis* E555 in response to 12-bis-THA. The **(A)** high **(B)** low field regions of 1H-NMR spectra of the intracellular metabolome of untreated *B. thailandensis* E555 or following incubation with 12-bis-THA **(C)** PCA analysis of showing the separation of *B. thailandensis* E555 metabolomes between components one and two. Blue, red and green clusters represent the metabolite profiles of untreated bacteria, or those treated with 0.1X MIC or 1.0X MIC of 12-bis-THA, respectively. **(D)** Identification of significant shifts in metabolite abundance determined using a one-way ANOVA where *p*-values < 0.05 are considered statistically significant (left to right: doublet at 1.18 ppm, betaine, glutamate, lysine, succinate, trehalose and uracil).

The proteomic data showed that 12-bis-THA caused a change in the abundance of proteins associated with central metabolism in *B. thailandensis*. Therefore, we hypothesized that these changes would lead to demonstrable changes at a metabolomic level. To test this, we performed NMR-based metabolomic analysis on cell pellets retained from the growth experiment shown in Figure 2A. Spectra recorded from these samples allowed for the identification of amino acids (including glutamate, valine, serine, tyrosine), osmolytes (including glucose, trehalose, glycine betaine), organic acids (including fumarate, succinate, pyruvate), cofactors (NAD+), nucleosides (including guanosine, adenosine, uracil) and metabolic intermediates and end-products (including ATP, ADP, AMP, 3-hydroxybutyrate; Figures 3A,B). PCA analysis showed considerable overlap between the metabolome of bacteria incubated with 0.1X MIC and the untreated control (Figure 3C). The metabolome of bacteria incubated with 1.0X MIC was distinct from the other groups but did show modest overlap with the untreated control. Univariate analysis showed the levels of lysine had increased and those of betaine, trehalose, glutamate, uracil, succinate and an unknown compound characterized by a doublet at 1.18 ppm had reduced (Figure 3D; Supplementary Figure S1) in response to 1.0X MIC.

### 3.5. Differential abundance of metabolites associated with osmotic protection and central metabolism

**Figure 4:**
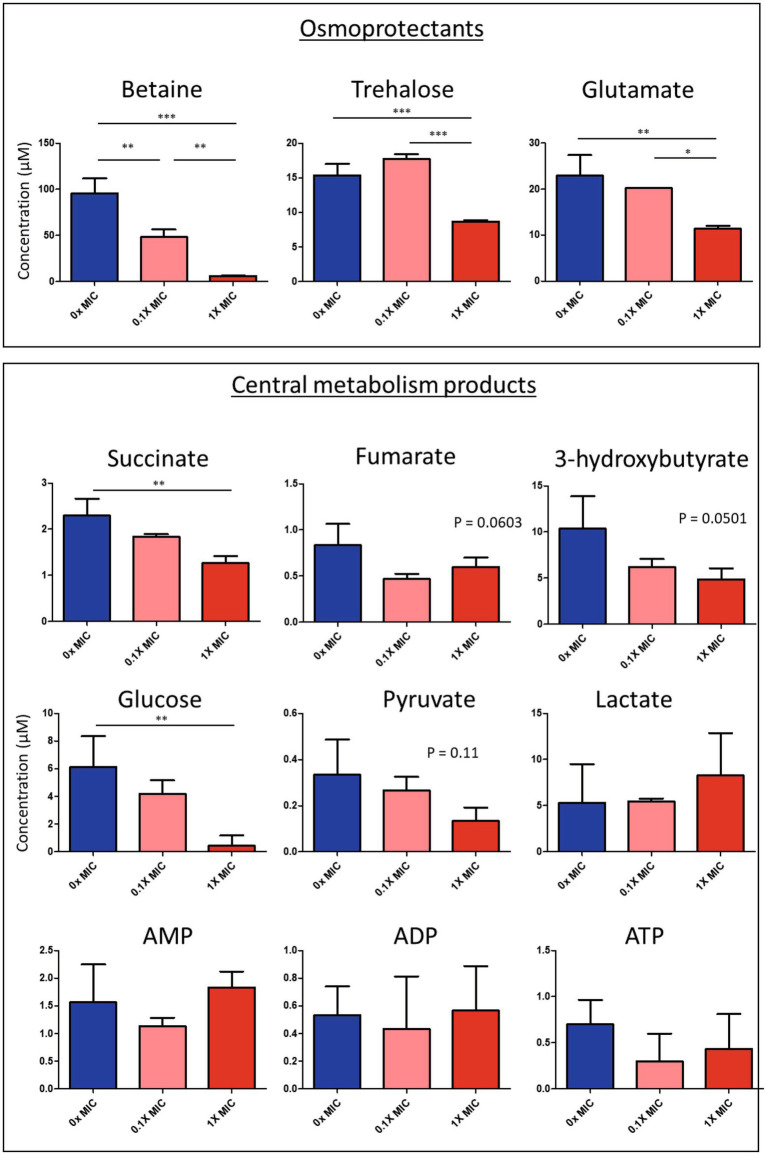
Intracellular concentration of select metabolites isolated from *B. thailandensis* E555, following treatment with 0.1X or 1.0X MIC of 12-bis-THA. Significance was denoted using *p < 0.05; **p < 0.01; ***p < 0.001. Data representative of three independent replicates.

Univariate analysis showed significant downregulation of succinate in response to 1.0X MIC of 12-bis-THA (Figure 4). Similarly, the cytoplasmic concentration of fumarate was also reduced however this was not statistically significant (Figure 4). 3-hydroxybutyrate, a ketone body that can be converted to acetyl-CoA, was also downregulated in response to 1.0X MIC. Glycolysis products were also detected in the metabolomic data set. Cytoplasmic glucose was significantly reduced under the same conditions. Additionally, the concentrations of cytoplasmic pyruvate were also reduced in response to 12-bis-THA while those of lactate increased although none reached significance. AMP, ADP and ATP were identified in the NMR spectra however none was differentially abundant following exposure to either 12-bis-THA concentration. The NMR spectra showed that 12-bis-THA induced changes in the cytoplasmic concentration of osmolytes. Statistical analysis showed that betaine (also known as trimethylglycine or glycine betaine) was the only metabolite to be downregulated in response to both 0.1X and 1.0X MIC (Figure 4). The osmolytes, glutamate and trehalose, were also downregulated however this was only observed in response to 1.0X MIC (Figure 4).

## 4. Discussion

In this study, we have investigated the antimicrobial activity of the novel molecule, 12-bis-THA against the surrogate organism, *B. thailandensis*. 12-bis-THA is a membrane-active molecule for which internalization is driven through electrostatic interactions with anionic membrane components such as cardiolipin (Marín-Menéndez et al., 2017) and LPS (Montis et al., 2020). *Burkholderia* species are intrinsically resistant to cationic membrane-active molecules through the modification of the lipid A component of LPS to reduce net anionic charge (Loutet and Valvano, 2011). 12-bis-THA does not appear to be affected by this and inhibited the growth of capsulated and unencapsulated strains of *B. thailandensis* at 2–8 μg/ml. Whilst no difference in MIC was observed between the two strains, a time-kill study showed that capsulated *B. thailandensis*, E555, was less susceptible to 12-bis-THA. To understand the protective role of the capsule in this strain of *B. thailandensis*, membrane permeabilisation and microscopy studies were performed. The membrane permeabilisation study showed that the capsulated strain E555 was less susceptible to membrane leakage compared to the unencapsulated strain however this was only observed at high concentrations of 12-bis-THA. This suggests that lysis is not the antimicrobial mechanism of 12-bis-THA at MIC concentrations.

Confocal microscopy showed that the blue fluorescent signal of 12-bis-THA was observed in all bacterial cells regardless of the strain, suggesting that its internalization is unaffected by the presence of the capsule. However, it is important to note that this assay requires the use of high concentrations of 12-bis-THA to exploit its intrinsic fluorescence, which may mask any difference in internalization kinetics observed at MIC concentrations. Regardless of bacterial strain, the fluorescence of 12-bis-THA was observed as a diffuse signal in the cytoplasm or associated with the cell membrane. Some areas of the cytoplasm were less bright and these may correspond to nucleoids, suggesting that though 12-bis-THA is a known intercalator it does not readily penetrate the densely packaged nucleoprotein complexes. However, further studies are required to investigate whether 12-bis-THA binds to DNA or RNA within the cell and whether this contributes to the antimicrobial activity. Bacterial cells incubated with 12-bis-THA also displayed a reduction in membrane stain resolution. The membranes were stained with FM4-64, a lipophilic cation that becomes embedded within the cytoplasmic membrane of bacterial cells (Mileykovskaya and Dowhan, 2009). The loss of resolution likely occurred due to interactions between 12-bis-THA and phospholipids within the cytoplasmic membrane resulting in the displacement of the dye. Cardiolipin is located exclusively in the inner membrane of eukaryotic mitochondria and the cytoplasmic membranes of bacteria, including *Burkholderia cenocepacia* (Sohlenkamp and Geiger, 2016), where it supports the function of some membrane bound proteins including respiratory complexes (Lin and Weibel, 2016). Due to its conical structure, it accumulates in areas of great curvature such as the poles or septa of bacteria, where it has been shown to form localized membrane domains (Mileykovskaya and Dowhan, 2000; Kawai et al., 2004). The confocal images suggest the polar accumulation of 12-bis-THA and the loss of resolution of the membrane stain. This may occur as these areas are rich in cardiolipin however this requires further investigations to confirm this.

Integrated, untargeted ‘omics approaches are a useful toolbox to provide a holistic overview of bacterial changes in response to stimuli and, as such, we performed a proteomic-metabolomic study to investigate the response of *B. thailandensis* E555 to 12-bis-THA. A TMT-based proteomics study was performed, and peptide reads mapped to the UNIPROT proteome of *B. thailandensis* E264. A consequence of this was that no changes in the abundance of proteins involved with the production and export of the capsular polysaccharide could be observed. TMT-based proteomics has been used previously to investigate the response of *B. thailandensis* E264 to a sub-inhibitory concentration of trimethoprim. 2,463 proteins and 2,524 proteins were identified at 1.0 and 5.0 OD_600_ in this study representing a proteome coverage of 43 and 45%, respectively (Li et al., 2020). These observations are in line with our findings, as we identified 3,150 proteins to give a proteome coverage of 56.6%.

NMR-based metabolomics has previously been used to investigate bacterial stress adaption in response to environmental stimuli or antimicrobial exposure (Hoerr et al., 2016). Taking a similar approach, we identified 45 metabolites in the cytoplasm of *B. thailandensis* which is comparable with a study that identified 25 metabolites in the cytoplasm of *B. cenocepacia* (Moreira et al., 2016). Our complementary data sets analyzing the proteome and metabolome of E555 treated cells will provide insight into the metabolic pathways affected by 12-bis-THA. Statistical analysis showed little variation in the metabolomes and proteomes of untreated cultures and those incubated with 0.1X MIC of 12-bis-THA. This may be due to the following reasons. Stringent statistical parameters were selected to limit the number of false positives which may increase the number of false negatives. Also, the treatment of 0.1X MIC 12-bis-THA was selected to represent a sub-inhibitory concentration which had been determined by MIC assay. However, MIC assays predict the inhibition of bacteria at a starting density of 10^5^ CFU/ml of bacterial cells whereas the bacteria in the growth curve study were at a bacterial density of approximately 10^10^ CFU/ml. 139 proteins increased in abundance and 671 were decreased in abundance following exposure to 1.0X MIC. The observed fold changes of differentially abundant proteins were comparable to those observed in a similar study (Li et al., 2020). Analysis of the metabolomic data identified one metabolite out of the 45 detected that increased in abundance and six that were reduced in response to 1.0X MIC.

Interestingly, lysine biosynthesis is known to occur in adaptation to stress (Isogai and Takagi, 2021). We observed a 4-fold increase in lysine levels in response to 1X MIC (Supplementary Figure S1) suggesting that *B. thailandensis* is adjusting to the new conditions by actively accumulating intracellular lysine. This phenomenon has been observed in yeasts and other microbe types highlighting the potential protective role of lysine utilization by microorganisms as an anti-stress mechanism (Neshich et al., 2013; Olin-Sandoval et al., 2019). This possibility offers exciting avenues for further research.

The cytoplasmic concentrations of ATP, pyruvate, and fumarate were not reduced however, this may be an artifact as the concentrations of these compounds were at the limit of detection of the assay where it is difficult to distinguish between authentic values and background noise. Thus, our untargeted proteomic and metabolomic studies have identified differentially abundant pathways and compounds to provide insight into the mechanism of action of 12-bis-THA against *B. thailandensis*.

Our previous evidence suggests that 12-bis-THA crosses the Gram-negative membrane, at least in part, due to electrostatic interactions with negatively charged membrane components such as cardiolipin (Marín-Menéndez et al., 2017) and LPS (Montis et al., 2020). This process may cause transient destabilization of the bacterial envelope resulting in the leakage of cytoplasmic constituents. This was supported by the findings of the NMR study which showed a reduction in the osmolytes glucose, betaine, trehalose and glutamate in response to 12-bis-THA which were likely affected by cytoplasmic leakage. In *B. cenocepacia*, betaine, trehalose, and glutamate were all important in responding to hyperosmotic stress and provide protection possibly against hypertonicity-induced cell shrinkage (Behrends et al., 2011). The loss of these compounds in response to 12-bis-THA may occur due to the permeabilisation of the *B. thailandensis* membrane, causing passive osmotic balancing to protect from changes in cell turgor.

The proteomic and metabolomic data both identified changes in central metabolism in response to 12-bis-THA, including the downregulation of phosphoglucomutase, 6-phosphofructokinase, and phosphoglycerate mutase which are enzymes associated with glycolysis, a conserved pathway that is responsible for the conversion of glucose to pyruvate. Similarly, the metabolomic data showed that both pyruvate and glucose were also reduced under the same conditions suggesting a reduction in glycolytic flux. Glycolysis breaks down a single glucose monomer to produce two ATP, two NADH, two H^+^ and two pyruvate molecules, the latter of which is converted into acetyl-CoA which is essential for the TCA cycle. The proteomics data set showed strong induction of the four enzymes comprising the β-oxidation cycle (BTH_I0564 – BTH_I0567). This pathway is responsible for the catabolism of long-chain fatty acids and produces FADH_2_, NADH and acetyl-CoA per completion of the cycle. This data suggests that 12-bis-THA causes a shift from glucose metabolism to fatty acid metabolism. A reduction in glycolysis may occur if glucose reserves were depleted in response to osmotic balancing. Induction of β-oxidation may then accommodate the suppression of glycolysis and support the maintained activation of TCA cycle and the electron transport chain (ETC).

The ETC is a series of redox reactions in which electrons are transferred from high-energy cytoplasmic donors (e.g., NADH and FADH_2_) to electron acceptors (e.g., cytochrome c). This is coupled with the transfer of protons from the cytoplasm to the periplasm to produce a proton gradient which is then exploited by processes such as ATP synthesis and flagella-based motility. The proteomic data set showed that 12-bis-THA induced changes in the ETC, as proteins associated with respiratory complex III (cytochrome c reductase) and respiratory complex V (ATP synthase) were downregulated. Cytochrome c reductase is responsible for the oxidation and recycling of ubiquinol to reduce periplasmic cytochrome c and to release protons into the periplasm (Cramer et al., 2011). Three protein components of this system (PetA, PetB, and PetC) were identified and were all downregulated. The data also showed a downregulation of ATP synthase subunits. ATP synthase is responsible for trafficking protons from the periplasmic space to the cytoplasm and driving the formation of ATP from ADP and inorganic phosphate. It is comprised of two components: (I) a membrane-bound domain, termed F_0_, which drives the translocation of protons from the periplasm to the cytoplasm and (II) a cytoplasmic facing component, termed F_1_, that is responsible for synthesizing ATP from ADP and inorganic phosphate (Nath, 2002). Subunit b acts as an anchor for the F_1_ domain and was the only F_0_ protein to be downregulated. Conversely, all of the detected F_1_ domain components were significantly downregulated, implying that 12-bis-THA inhibits cytochrome c reductase and ATP synthase. This is broadly consistent with the mechanism of its parental molecule, dequalinium which is a known inhibitor of mitochondrial respiratory complex III (Betosludtsev et al., 2017), in addition to eukaryotic and prokaryotic respiratory complex V (Hong and Pedersen, 2008).

Both ATP synthase and cytochrome c reductase are important proteins that contribute to the formation of ATP via ETC. As the proteomic dataset showed a reduction in the protein subunits of these enzymes, we anticipated a changes in the cytoplasmic concentrations of ATP and ADP concentration in response to 12-bis-THA. However, though both ATP and ADP were detected, neither changed in abundance. This may occur as both metabolites were identified near the limit of detection. LC/MS metabolomics and plate-based ATP quantification assays would be a more sensitive way to identify concentration and time-dependent changes in ATP concentration in response to 12-bis-THA. The mechanism by which 12-bis-THA inhibits respiratory complexes is currently unknown. One potential mechanism is that 12-bis-THA may uncouple respiratory complexes by binding cardiolipin which has been shown to bind and facilitate the activity of respiratory chains III and V in mitochondria (Mühleip et al., 2019). However, this is unlikely to be the sole cause of respiratory chain depression as cardiolipin has also been shown to support the activity of respiratory complex I (NADH dehydrogenase; Dancey and Shapiro, 1977) which was not affected in our study (data not shown). Alternatively, the binding of cardiolipin within the cytoplasmic membrane by cationic 12-bis-THA may dissipate membrane potential, which has been observed for its parental molecule, dequalinium, in mitochondria (Bae et al., 2018). Accumulation of 12-bis-THA within the inner membrane may cause depolarization of the membrane by interrupting the chemo-osmotic gradient, either through the leakage of protons across the membrane due to permeabilisation, or by neutralizing the charge gradient by the cationic headgroup of 12-bis-THA. This could prevent the re-uptake of protons, concomitantly suppressing the action of ATP synthase.

Loss of proton motive force (PMF) can be countered by increasing TCA flux. This has been shown through the addition of exogenous glucose (Su et al., 2018) or fumarate (Meylan et al., 2017) to the growth medium of *Pseudomonas aeruginosa*. PMF might be stimulated through the suppression of the glyoxylate cycle which intersects the TCA cycle and bypasses the oxidation of α-ketoglutarate and succinyl coenzyme A. By doing so, this retains two carbons in the form of acetyl-CoA which would otherwise be converted to CO_2_ (Cronan and Laporte, 2005), however this is performed at the loss of two NADH molecules. Suppression of this pathway would produce more NADH which could be used to stimulate PMF. The proteomic data showed that AceA and AceB, the enzymatic components of the glyoxalate cycle, were downregulated in response to 12-bis-THA. Additionally, α-ketoglutarate dehydrogenase (SucAB) was upregulated under the same conditions. The NMR data showed a depletion of succinate and fumarate which is consistent with 12-bis-THA increasing TCA flux. Oligomycin and dicyclohexylcarbodiimide are respiratory uncouplers that prevent the movement of protons from the periplasm to the cytoplasm inhibiting the formation of ATP due to the absence of PMF. Dequalinium has also been shown to dissipate membrane potential in mitochondria (Bae et al., 2018), thus we postulate that flux through the TCA cycle is increased to provide high-energy compounds to the ETC to counter membrane depolarization. Future studies are required to investigate changes in membrane potential in *Burkholderia* species in response to 12-bis-THA.

Melioidosis is difficult to treat as it requires a combination of intravenously administered antimicrobials followed by long course of oral therapy. Although the resistance to clinically relevant therapeutics is low, cases have been reported and it is therefore important to identify new antimicrobial candidates that are active against *B. pseudomallei*. In this study, we have investigated the stress response of *B. thailandensis* strain E555 to 12-bis-THA with a view to understanding its antimicrobial action and to determine whether it has merit for further investigation. We have shown that it alters pathways of the central metabolism in two ways: (1) through the inhibition of ATP synthase, potentially due to the dissipation of PMF either through membrane permeabilisation or due to interruption of the chemo-osmotic gradient; (2) compensatory changes the TCA cycle and the glyoxylate cycle possibly to stimulate PMF.

Future studies are ongoing to provide further insight in the mechanism of action of 12-bis-THA. We hypothesize that 12-bis-THA inhibits ATP formation through the dissipation of PMF. ATP (Lemasters and Hackenbrock, 1978) and PMF (Panta et al., 2019) could be quantified by using plate-based assays to support the systems biology data sets. Additionally, our data shows that 12-bis-THA particles can induce changes in central metabolism that are associated with increased susceptibility to aminoglycosides in *P. aeruginosa*. Therefore, we hypothesize that 12-bis-THA might synergize with aminoglycosides, such as tobramycin, against *Burkholderia* species which are typically resistant to these compounds. Further studies are required to investigate the potential of 12-bis-THA as an aminoglycoside adjuvant to counter *Burkholderia* infections.

## Data availability statement

The mass spectrometry proteomics data have been deposited to the ProteomeXchange Consortium via the PRIDE partner repository with the dataset identifier PXD041456.

## Author contributions

AP: formal analysis, study design, data acquisition, and writing – original draft. SB: study design, methodology development, and writing – editing. GG: method development, study design, data acquisition (NMR), and writing – editing. CM and SH: study design, supervision, and writing – drafting and editing. MM: study design, funding acquisition, project management, supervision, and writing – drafting and editing. All authors contributed to the article and approved the submitted version.

## Funding

This project was funded by UK Ministry of Defense.

## Conflict of interest

MM was previously the CSO of Procarta Biosystems, the developer of 12-bis-THA.

The remaining authors declare that the research was conducted in the absence of any commercial or financial relationships that could be construed as a potential conflict of interest.

## Publisher’s note

All claims expressed in this article are solely those of the authors and do not necessarily represent those of their affiliated organizations, or those of the publisher, the editors and the reviewers. Any product that may be evaluated in this article, or claim that may be made by its manufacturer, is not guaranteed or endorsed by the publisher.

## Supplementary material

SUPPLEMENTARY FIGURE S1 Intracellular concentration of metabolites isolated from *B. thailandensis* E555, following treatment with 0.1X or 1.0X MIC of 12-bis-THA. Significance was denoted using **p* < 0.05; ***p* < 0.01; ****p* < 0.001. Data representative of three independent replicates.
