# Supplementary figures and images for: Using a multi-omic approach to investigate the mechanism of 12-bis-THA activity against Burkholderia thailandensis
Source: Front Microbiol. 2023 May 12;13:1092230. doi: 10.3389/fmicb.2022.1092230 (PMC10213367; doi:10.3389/fmicb.2022.1092230)

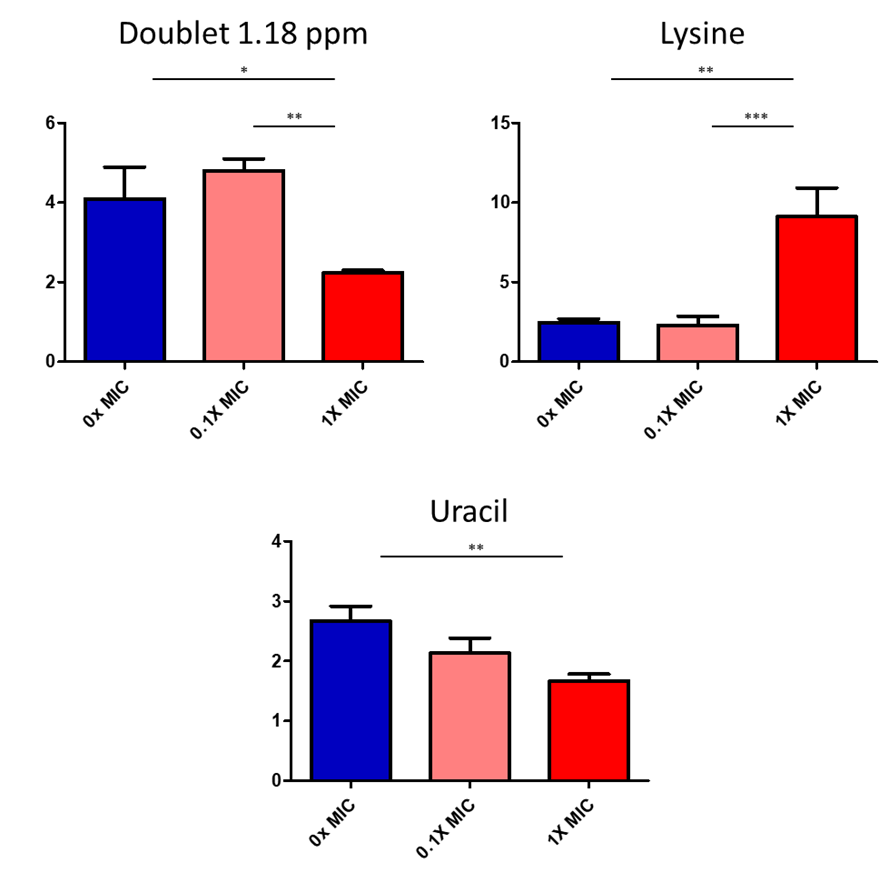

Supplement: SUPPLEMENTARY FIGURE S1 — Intracellular concentration of metabolites isolated from B. thailandensis E555, following treatment with 0.1X or 1.0X MIC of 12-bis-THA. Significance was denoted using *p < 0.05; **p < 0.01; ***p < 0.001. Data representative of three independent replicates. [file Image_1.png]
